# Supplementary figures and images for: Inflammasomes primarily restrict cytosolic Salmonella replication within human macrophages
Source: eLife. 2025 Mar 27;12:RP90107. doi: 10.7554/eLife.90107 (PMC11957546; doi:10.7554/eLife.90107)

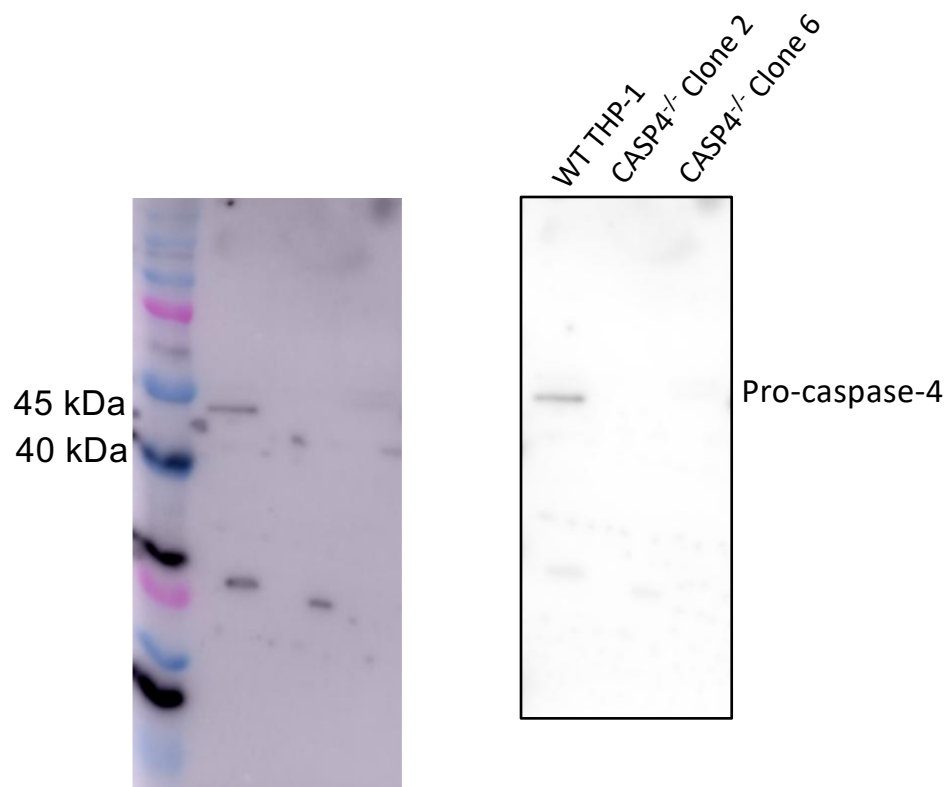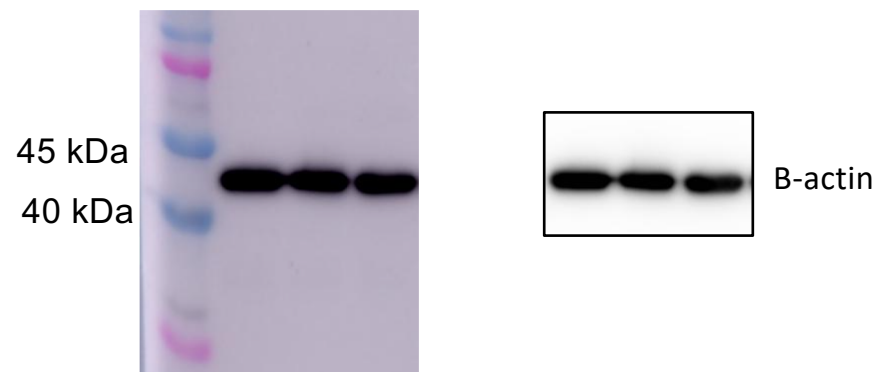

Supplement: Figure 2—figure supplement 1—source data 1. [file elife-90107-fig2-figsupp1-data1.pdf]
